# Supplementation with effective microorganisms in earthen ponds affects common carp growth and abundance of specific bacterial families

**DOI:** 10.1186/s12864-026-13093-z

**Published:** 2026-06-23

**Authors:** Michalina Jakimowicz, Katarzyna Sidorczuk, David Huyben, Falk Hildebrand, Łukasz Napora-Rutkowski, Piotr Hajduk, Marek Sztuka, Magda Mielczarek, Dawid Słomian, Laura Jarosz, Joanna Szyda

**Affiliations:** 1 Department of Genetics, Wroclaw University of Environmental and Life Sciences, Kozuchowska 7, Wroclaw, 51-631 Poland; 2 Quadram Institute, Norwich Research Park, Norwich, NR4 7UQ UK; 3 Earlham Institute, Norwich Research Park, Norwich, NR4 7UQ UK; 4 Department of Animal Biosciences, University of Guelph, 50 Stone Road East Guelph, Guelph, ON N1G 2W1 Canada; 5 Institute of Ichthyobiology and Aquaculture, Polish Academy of Sciences, Kalinowa 2, Chybie, 43-520 Poland; 6 Department of Cattle Breeding, National Research Institute of Animal Production, 1 Krakowska Street, Balice, 32-083 Poland; 7 Department of Biomedical Engineering, The Biostatistic Group, Wroclaw University of Science and Technology, Plac Grunwaldzki 13, Wroclaw, 50-378 Poland

**Keywords:** 16S rRNA, Aquaculture, Gut, Metatranscriptome, Microbiome, Sediment, Water

## Abstract

**Background:**

Effective microorganisms are increasingly explored in aquaculture to improve fish health and growth without leaving harmful residues. However, their efficacy in real-world pond environments remains poorly understood. Here, we conducted a 103-day field experiment to assess the effects of supplementation with two effective commercial microorganism products on the microbial communities and growth performance of common carp (*Cyprinus carpio*).

**Background:**

Effective microorganisms were added to the feed and directly to the pond water. Microbial diversity was analysed via 16 S rRNA and whole-genome shotgun sequencing across three environments: water (three time points), sediment (two time points), and fish intestine (one time point) from 25 experimental ponds. Bioinformatics processing was performed using the QIIME2 and MG-TK pipelines with taxonomic classification based on the SILVA database. The results showed that although supplemented bacterial families did not establish significantly in pond environments, fish exposed to specific effective microorganism treatments showed improved growth metrics.

**Conclusions:**

These findings suggest that effective microorganisms can increase carp growth in aquaculture without significantly altering the resident microbial communities, suggesting a promising residue-free alternative to traditional additives in aquaculture.

**Supplementary Information:**

The online version contains supplementary material available at https://doi.org/10.1186/s12864-026-13093-z.

## Introduction

To meet the growing market demand for aquaculture products and maximise production, feed additives such as growth promoters, vitamins, and antibiotics are commonly used in aquatic animal feed to combat pathogens and improve disease resistance [1]. Growing concerns over antibiotic residues in aquaculture products have driven the demand for additive-free alternatives. Therefore, new strategies for improving fish health and increasing productivity are being explored in aquaculture. Effective microorganisms are living microorganisms that confer beneficial effects to the host when administered in adequate amounts [2]. Their use in aquaculture has shown promise, as a large number of genes and their products provided by microbial communities residing in water, sediment, and hosts (fish) are likely to impact aquaculture production, as summarised by [1, 3, 4]. The direct impacts include: (i) improving the general health of fish, enhancing their digestive enzymes, (ii) enhancing feed efficiency, thus resulting in overall better growth performance, (iii) increasing resistance against infections, and (iv) improving reproduction. Aquaculture production is also likely to be indirectly impacted by improving water quality, reducing abundance of pathogenic microbial species, providing nutrients for fish, and breakdown of organic waste. In this context, effective microorganisms offer a safe alternative to antibiotics, and supplementing them in water and feed has emerged as a strategy to influence microbial composition.

The common carp (*Cyprinus carpio*) is an important aquaculture species in many countries, especially in Eastern and Central Europe. In 2020, it was one of the most farmed fish species globally [5], and an increase in demand is predicted in the future. Although, as reviewed by [6], research on the impact of effective microorganism supplementation in common carp has been intensively carried out for almost two decades, there has been little research related to the practical, on-farm (i.e. not laboratory) application of effective microorganism products, especially those that are mixed microorganism communities rather than specific bacterial species. The effects of feed supplementation with particular bacterial species, including *Lactobacillus casei* [7], *Lactobacillus fermentum* [8], and *Pediococcus acidilactici* [9] on the immune response and disease resistance of carp have previously been studied. Furthermore, the effects of *Lactobacillus acidophilus* have been analysed in the context of multiple traits in common carp, including growth performance and immune response [10, 11, 12]. reported a positive effect of *Lactobacillus acidophilus* on the growth rate and immune response of carp [13]. demonstrated an improved immune response due to supplementation with *Enterobacter asburiae*. Beneficial effects on the digestive system after *Lactobacillus rhamnosus* supplementation were reported by [14], whereas [15] reported an overall increase in resistance to disease after supplementation of a carp diet with *Bacillus subtilis* [16]. studied the general effect of effective microorganisms supplementation on carp growth performance. However, in all the studies mentioned above, water tanks were used as laboratory conditions and to our knowledge, the only reported field experiment involving effective microorganism supplementation of the common carp was described by [17]. It is important to realise that although laboratory-based studies in tanks provide a valuable and cost-effective initial experimental setup, they miss a considerable proportion of biological and environmental factors that are present in the field. Therefore, experiments conducted in real-world, farm settings provide more representative results and have greater implications for practical applications in aquaculture.

Our study was conducted as a field experiment in earthen ponds to reflect practical fish rearing conditions while investigating how supplementation with effective microorganisms affects microbial diversity, the composition of the microbiome, and its dynamics over time. Our objectives were to assess the effects of supplementation across three different environments: (i) pond water, (ii) pond sediment, and (iii) fish gut, as well as its impact on fish growth performance. These findings were further enhanced by studying the effects of microbial supplementation on fish growth performance. We supplemented common carp (*Cyprinus carpio*) feed and ponds with effective microorganisms in a 103-day experiment and evaluated the microbial composition. Microbiome composition was identified by sequencing the V3 and V4 hypervariable regions of the gene encoding the 16 S rRNA ribosomal subunit, and the metatranscriptome of water was assessed via whole-genome shotgun reads.

## Materials and methods

### Experimental design

**Fig. 1:**
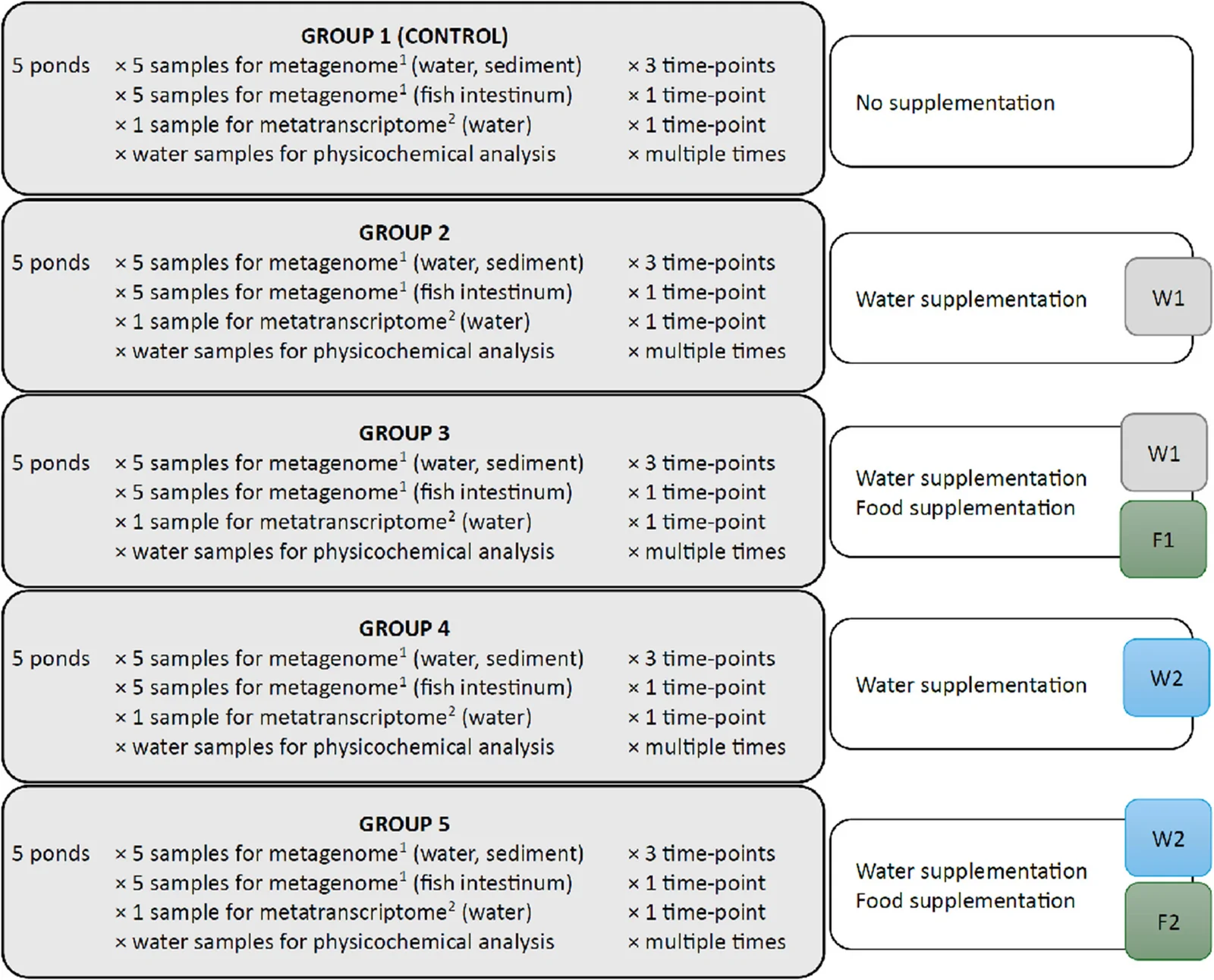
The experimental design included five ponds without - (Group 1), with water - (Groups 2 and 4) as well as with water and feed - (Groups 3 and 5) supplementation. Water supplements are represented by W1 and W2, whereas feed supplements are represented by F1 and F2. (1) 16 S rRNA gene sequencing and (2) whole-sample RNA sequencing (metatranscriptome)

The facility consisted of 25 earthen ponds in the Gołysz experimental station of the Polish Academy of Sciences, each covering an area of 600 m^2^ and supplied with freshwater from the Vistula River to maintain consistent physicochemical properties of the water. The experiment spanned the entire fish production season, from May to October 2022, including a preliminary period of pond preparation from March to May. Each pond was stocked with 500 ± 10 common carp fry (1.0 g ± 0.2 weight) originating from the same hatchery. The experimental ponds were classified into five groups, each consisting of five ponds that served as biological replicates. Group 1 (control) received no supplementation. Group 2 included ponds in which water was supplemented with W1 without feed supplementation. Group 3 included ponds supplemented with W1 water supplement and F1 feed supplement. Group 4 included ponds in which water was supplemented with W2 without feed supplementation. Group 5 included ponds supplemented with W2 water supplement and F2 feed supplement (Fig. 1). According to the manufacturer, the W1 water supplement included *Lactobacillus sp*., three species of *Bifidobacterium sp*., *Carnobacterium divergens*, *Streptococcus salivarius*, and *Saccharomyces cerevisiae*. The feed supplement F1 contained all the above species and *Lactobacillus delbrueckii*, *Bacillus subtilis*, *Rhodopseudomonas palustris*, and *Rhodobacter sphaeroides*. The water supplement W2 and the feed supplement F2 consisted of *Lactobacillus plantarum*,* Lactobacillus casei*, and *Saccharomyces cerevisiae*, along with several species that were confidentially protected by the manufacturer. Water supplements were applied to the bottom (20 l/ha) and water surface (20 l/ha) four times during the first month of the experiment before the first water and sediment sampling. The feed supplement was mixed with the commercial complete grower feed Aller Classic Vitamax (Aller Aqua, Poland) and applied twice a week throughout the experiment at a concentration of 2 l/t. The grower was composed of fish meal, triticale grain distillers, hydrolysed feather meal, poultry blood meal, poultry meal, rapeseed oil, sunflower protein concentrate, and monoammonium phosphate. Feed supplements W1 and W2 were added directly to a portion of the fish feed at a concentration of 2 l/t of feed, mixed in a bucket for 1 min, and then applied to the water of the experimental ponds. The fish were fed twice a week throughout the experiment, with a daily feeding rate of 5% of their body weight. The daily feed ratio was calculated by assuming a feed conversion ratio of 1.5 for common carp.

### Sample preparation

#### Sample collection

**Fig. 2:**
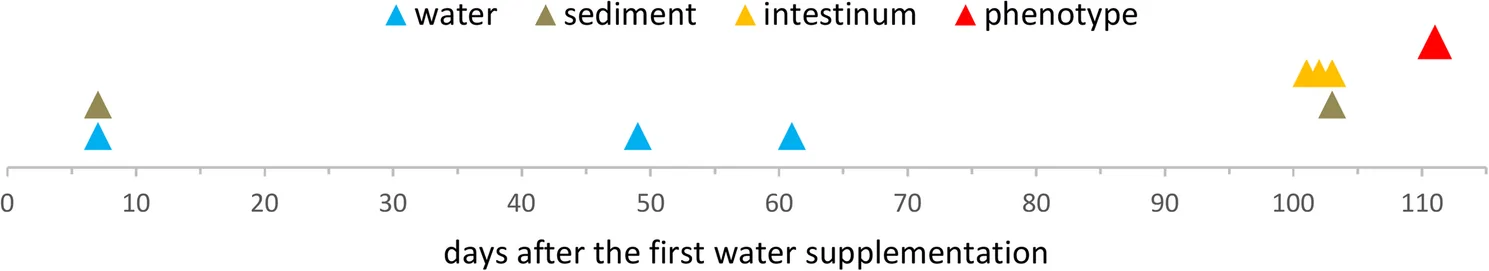
Timing of data collection

Three water samples were collected from each pond three times during the experiment, specifically at the beginning, seven days after the first water supplementation (T1), at the mid time-point 42 days after the 1st sampling (T2), and at the last month of the experiment, 54 days after the 2nd sampling (T3). Sediment samples were collected twice, at the beginning and during the last month of the experiment, that is, 96 days after the 1st sampling. Moreover, between days 101 and 103 after the beginning of the experiment, intestinal samples were collected from five common carp from each pond (Fig. 2).

Water samples were collected from the pond shoreline at an approximate depth of 0.5 m, with each sampling site using a 2 m long and 1000 ml volume sampling pole. Three sampling sites were selected from the middle of the three shorelines of each pond. From each sampling site, equal volumes of water were collected, mixed, stored in a 500 ml PEHD sterile bottle on ice, and transported to the laboratory, where they were stored at -20 °C until filtration. Microorganisms were collected by filtering approximately 500 ml of water through a sterile mixed cellulose ester (MCE) membrane with a pore size of 0.22 μm (47 mm diameter; ChemLand, Poland). All filter membranes were stored at -80 °C until DNA extraction. Sediment samples were collected using a three-point sampling method. The sediments were sampled using a corer made of 5.5 cm diameter metal tubing attached to a 2 m long wooden pole. The samples were collected from the surface of the collecting corer using a sterile metal spoon and a 50 ml sterile Falcon tube. Approximately 10 g of sediment was collected from three different sampling sites (the same as water was collected) in each pond and mixed immediately after collection to form a single sample. Fish were first anesthetized with 100 mg/l of MS-222, then another dose of MS-222 was administered to achieve a lethal concentration of 500 mg/l. Death was confirmed by transection of the spinal cord with a sharp instrument. After euthanasia, the anterior part of the intestine was dissected, and gut contents were squeezed out and collected for microbiome sequencing.

#### 16 S rRNA sequencing

Commercial DNA isolation kits dedicated to environmental samples were used for the isolation of DNA. The PowerSoil^®^, DNeasy PowerWater Kit and the QIAamp PowerFecal Pro DNA Kit (QIAGEN^®^, Germany) were used for sediment, water, and intestinal microbiota, respectively. The quality and concentration of DNA were assessed using gel electrophoresis and fluorometry. DNA samples were normalised to a concentration of five ng/µl. Then, 20 cycles of PCR were performed using primers that contained tag sequences. Electrophoresis of the PCR products was performed on a 1.5% agarose gel (Invitrogen, cat. no. 16500500) stained with GelGreen dye (Biotium cat. no. #41004). The samples were loaded with O range G loading dye (6X) (Thermo Scientific, cat. no. J60562.AC). The remaining 9 µL of samples were supplemented with MQ water to 20 µL and purified using the SPRI method, using the KAPA Pure Beads reagent (KAPA Biosystems cat. no. 07983271001) at a sample-to-reagent ratio of 1:0.8. Libraries were constructed from the prepared amplicons by incorporating adapters IDT for Illumina Nextera DNA UD Indices Set A (96 indices, 96 samples) (Illumina, cat. no. 20027213) in eight PCR cycles. KAPA HiFi HS RM was used (KAPA Biosystems cat. no.795893500) according to the manufacturer’s protocol. The libraries obtained were collected in 11 pools of 13–24 libraries each. The pools were subjected to fragment length control using the Tape Station 2200 analyser and a set of High Sensitivity D5000 Reagents (Agilent, cat. no. 5067–5593). The concentration of the libraries was determined by qPCR using the Kapa Library Quantification Kit, according to the manufacturer’s instructions (Kapa Biosciences, cat. no. KK4824). Fragment selection was carried out using Kapa Pure Beads in one-step purification. Sequencing of selected hypervariable regions (V3 and V4) of the gene encoding the 16 S subunit of a ribosome (16 S rRNA gene) was performed on an Illumina NovaSeq 6000 instrument using the NovaSeq 6000 SP Reagent Kit v 1.5 (500 cycles), with paired-end reads of 2 × 250 cycles according to the manufacturer’s instructions with 1% addition of the Phix control library (Illumina, code no. FC-110-3001).

#### Metatranscriptome sequencing

One water sample per pond was collected for metatranscriptome analysis on days 102–104 of the experiment. For RNA isolation, water samples were filtered using a sterile vacuum system through sterile MCE membranes with a pore size of 0.22 μm (47 mm diameter, ChemLand, Poland). The membranes were placed in sterile Eppendorf tubes, immediately frozen in liquid nitrogen to preserve the degradation of the sample RNA, and stored at -80 °C. The RNeasy PowerWater Kit (QIAGEN^®^, Germany) was used according to the manufacturer’s instructions. The RNA samples were qualitatively analysed using an Agilent TapeStation 2200 analyser and High Sensitivity RNA ScreenTape reagents (Agilent, no. 5067–5579), followed by eukaryotic ribodepletion using the NEBNext^®^ Globin & rRNA Depletion Kit (Human/Mouse/Rat) (New England Biolabs, cat. no. E7750X) and library construction was performed using the KAPA RNA HyperPrep Kit (KAPA Biosystems, cat. no. 8098107702) according to the manufacturer’s recommendations, starting with 255 ng of material. KAPA_UDI_Indexes adapters were used (96 indices, 96 samples) (KAPA Biosystems, cat. no. 08098140702). The RNA was fragmented at 85 °C for 6 min and ten cycles of enrichment by amplification were performed. The libraries obtained were subjected to fragment length control using the Agilent TapeStation 2200 analyser and the High Sensitivity D1000 Reagents set (Agilent, cat. no. 5067–5585). The library concentrations were determined by qPCR using the Kapa Library Quantification Kit (Kapa Biosciences, cat. no.KK4824). The manufacturer’s protocols were used. Sequencing was performed on an Illumina NovaSeq 6000 instrument using the NovaSeq 6000 SP Reagent Kit v 1.5 (500 cycles), with paired-end reads of 2 × 250 cycles according to the manufacturer’s instructions with a 1% addition of the Phix control library (Illumina, cat. no. FC-110-3001). The two control samples were discarded owing to poor data quality.

### Water quality, phenotypic data and fish survival rates

The common carp fry was harvested after 111 days. Phenotypic data was collected from 750 individuals (30 fish per pond) on the final day of the experiment (Supplementary table S1). Weight, total length (including tail fin), and predorsal height were measured. Weight was measured with an accuracy of 0.1 g. Length and height measurements were made on graph paper with an accuracy of 0.1 mm. In each pond, water quality indicators were determined seven times throughout the experimental period, every three weeks. The parameters included orthophosphate [mg/l], nitrite [mg/l], nitrate [mg/l], and ammonium [mg/l] concentrations were analysed using a spectrophotometer UV-1601PC (Schimadzu, Japan) and kits from Spectroquant^®^ and Supelco^®^ (Merck, Germany). One-way analysis of variance (ANOVA) was used for comparing the concentration of water quality indicators between experimental groups, separately at each sampling point. In case of significant between-group differences a Tukey’s HSD post-hoc test for pairwise comparisons was calculated. Two-sample t tests were used for each of the possible pairwise comparisons of group specific survival rates with the resulting nominal P values subjected to Bonferroni correction.

### Bioinformatic analysis

#### 16 S rRNA

The processing and analysis of 16S rRNA sequences were performed using QIIME2 software [18]. The processing pipeline involved: (1) assessment of raw read quality, (2) trimming sequences with quality scores below 30 from the 5’ and 3’ ends, (3) removing sequencing adapters, (4) removing sequences shorter than 200 bp, (5) merging paired-end reads, (6) additional quality filtering steps (quality filter q-scores), and (7) denoising (qiime deblur denoise-16 S). This pipeline yielded Amplicon Sequence Variants (ASVs) that were used to determine the microbial composition of the samples. Taxonomic assignment was completed using QIMME2 feature classifier, which implements a Bayesian classification model pretrained on 16 S rRNA data. All analyses following taxonomic assignment were performed using the SILVA (v.138.1) database [19] at the family level.

The alpha diversity metric quantifying microbial diversity within a pond was expressed by Shannon’s index: $\:H=-1\cdot\:\sum_{i=1}^{S}{p}_{i}ln\left({p}_{i}\right),\:$ where * S* represents the number of families present in a given pond and where $\:{p}_{i}$ is the frequency of the i-th family. The bacterial compositions of the water, sediment, and gut environments were expressed as PCA-based embedding and then visualised using the first two principal component vectors. For the computation of embeddings, the *prcomp* function from the *stats* library implemented in R [20] was applied with default parameters, using abundances normalised with Centered Log-Ratio (CLR) transformation: $\:CLR\left({x}_{i}\right)=ln\left(\frac{{x}_{ij}}{G\left({X}_{i}\right)}\right)$, where $\:{x}_{ij}$ represents the raw abundance of the *j*-th bacterial family within the *i*-th sample (fish or pond) and $\:G\left({X}_{i}\right)$ is the geometric mean of raw abundances representing sample *i* [21]. The CLR-transformed abundances of bacterial families in fish intestine, water and sediment are provided in Supplementary tables S2-S4.

Furthermore, we analysed the differences in the abundance of particular bacterial families using the DESeq2 package [22] implemented in R. For the gut samples, pairwise comparisons were made between the control (Group 1) and each experimental group (Groups 2, 3, 4, and 5). For environmental samples, comparisons were made at three (water) and two (sediment) time points. At the first time point, the control group (Group 1) was compared with all experimental groups combined (Groups 2–5). At the second and third time points, pairwise comparisons of gut samples were made between the control group (Group 1) and each experimental group (Groups 2, 3, 4, and 5). The bacterial family abundances were normalised using the default median-of-ratios normalisation implemented in DESeq2 [23]. The differential abundance was then expressed as the log_2_FoldChange (LFC) between samples, whereas hypothesis testing was based on the Wald test $\:W=\frac{{\widehat{LFC}^2}}{{\hat{\sigma}^2_{LFC}\:}}$, which under the null hypothesis of no differences in abundances asymptotically follows the standard normal distribution. The nominal P-values corresponding to each tested family were corrected for multiple testing using the Bonferroni approach.

Specifically for the supplemented families, their survival in water and sediment over the course of experiment was tested using a linear regression of the form $\:y={\beta\:}_{0}+{\beta\:}_{1}t$ where * y* represents the abundance of supplemented families and * t* is the time point class (T1, T2, T3). A separate linear regression was fit to water and sediment microbiome, separately for control ponds from Group 1, ponds supplemented with W1 (Groups 2 and 3) and ponds supplemented with W2 (Groups 4 and 5). The null hypothesis of $\:{\beta\:}_{1}=0.0$ was tested with a t test $\:=\frac{{\widehat{\beta\:}}_{1}}{{\hat{\sigma\:}}_{{\hat{\beta\:}}_{1}}}$, with the corresponding P values obtained based on the $\:{t}_{2}$ distribution. The linear regression was implemented in R via the *lm* function.

#### Metatranscriptome

Metatranscriptomes were primarily used to derive unbiased taxonomic compositions of water samples and were processed using the MG-TK pipeline [24] run in reference-based mode. Basic filtering, functional, and phylogenetic profiling were adapted from protocols used in [25, 26]. Filtering based on read quality was performed with a simple demultiplexer [27] by removing: (i) reads with estimated accumulated error exceeding 2.5 with a probability ≥ 0.01 [28], (ii) reads with more than one ambiguous position, or (iii) reads containing a homonucleotide run longer than 15 bp. Reads were trimmed if the base quality dropped below 20 in a window of 18 bases at the 3′ end or if the accumulated error exceeded 1. Filtered reads were profiled using the miTag approach [29]. The reads corresponding to the rRNA subunits were extracted using SortMeRNA [30] and searched against the SILVA (v.128) database [19]. Reads matching the database with E-values < 10^− 4^ were further filtered with custom Perl and C + + scripts using FLASH [31] to merge all matched read pairs. If read pairs could not be merged, single reads were interleaved so that the second read pair was reverse complemented and then sequentially added to the first read. Candidate interleaved or merged reads were then fine-matched to the SILVA database using Lambda3 [32]. To determine the identity of filtered reads based on Lambda3 matches, the lowest common ancestor (LCA) algorithm adapted from LotuS [27] was applied. The abundance of each taxon was normalised by dividing it by the total number of reads per sample to account for uneven sequencing depth across samples. Differential abundance of species was assessed in the same way as for 16 S data. Species-level abundances obtained from metatranscriptome analysis were provided in Supplementary tables S5 and S6.

### Modelling fish growth

The association between microbial family abundance in the gut and phenotypic measurements of individual fish (weight, height, and length) was analysed using two-way hierarchical ANOVA and linear penalised regression. In the ANOVA, an experimental group was used as the class variable, and ponds were modelled as nested effects. For linear regression, the data preprocessing step involved averaging the CLR-normalised abundance of each bacterial family within each pond over the five fish for which 16 S rRNA sequences were available. Then, the LASSO linear regression [33], which employs L1 norm regularisation, was fitted by minimising:

1 $$\sum_{i=1}^{N}{\left({y}_{i}-\sum_{j=1}^{M}{x}_{ij}{\beta\:}_{j}\right)}^{2}+\lambda\:\sum_{j=1}^{M}{\left|{\beta\:}_{j}\right|}_{}$$

where $\:{y}_{i}$ is the phenotypic measurement for the * i*-th fish, $\:{x}_{ij}$ represents the abundance of the * j*-th bacterial family averaged over the five ponds from experimental conditions corresponding to fish * i*, $\:{\beta\:}_{j}$ is the effect of the * j*-th family, $\:\lambda\:$ is the regularisation parameter, * N* is the number of individuals with phenotypes, and * M* is the overall number of bacterial families. Note that the hyperparameter $\:\lambda\:$ was set to 0.1 by comparing different $\:\lambda\:$ estimates from a cross-validation approach implemented in the *glmnet* R package.

## Results

### Water quality

For each water quality indicator (NH_4_, NO_2_, NO_3_, PO_4_) raw data was provided in Supplementary tables S9-S12 while means and standard deviation across ponds were given in Supplementary tables S13-S16. Across all experimental groups, the concentrations of water quality indicators largely followed a consistent pattern of peaking at the initial sampling and decreasing to the lowest levels by the final sampling. NO_3_ and PO_4_ exhibited higher variation across ponds from the same experimental group.

### Within-sample diversity

**Fig. 3:**
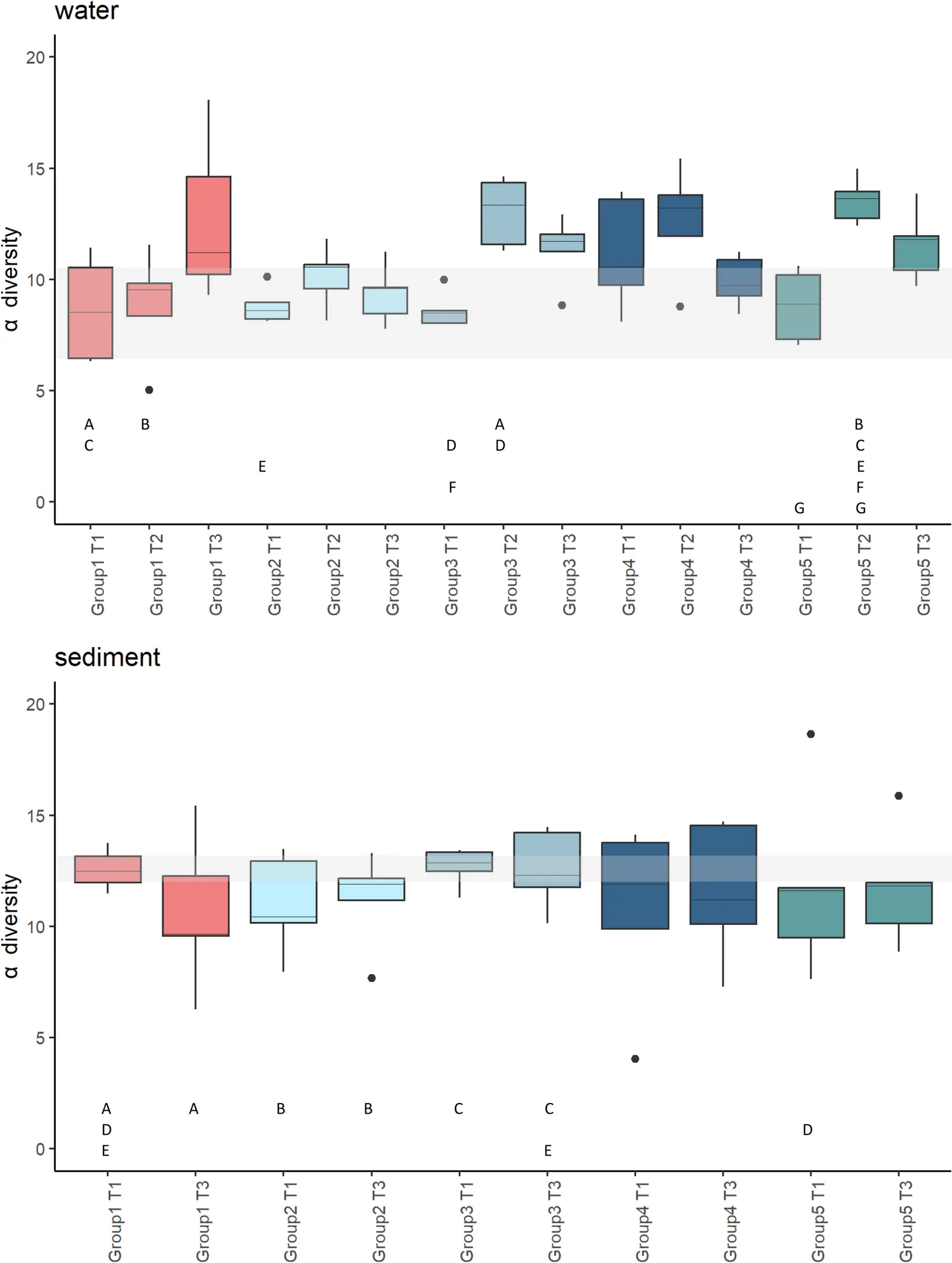
Alpha diversity (Shannon index) of water and sediment samples. T1 (day 5), T2 (day 47), and T3 (day 100) represent the consecutive sampling time points. Group 1 is the control group (5 ponds), Group 2 is experimental group W1 (5 ponds), Group 3 is experimental group W1-F1 (5 ponds), Group 4 is experimental group W2 (5 ponds), and Group 5 is experimental group W2-F2 (5 ponds), where F and W denote feed and water supplements, respectively. The shaded areas represent 75% of the variation in alpha diversity observed in control ponds at T1. Letters mark groups with significant (*P* < 0.05) pairwise - diversity

**Fig. 4:**
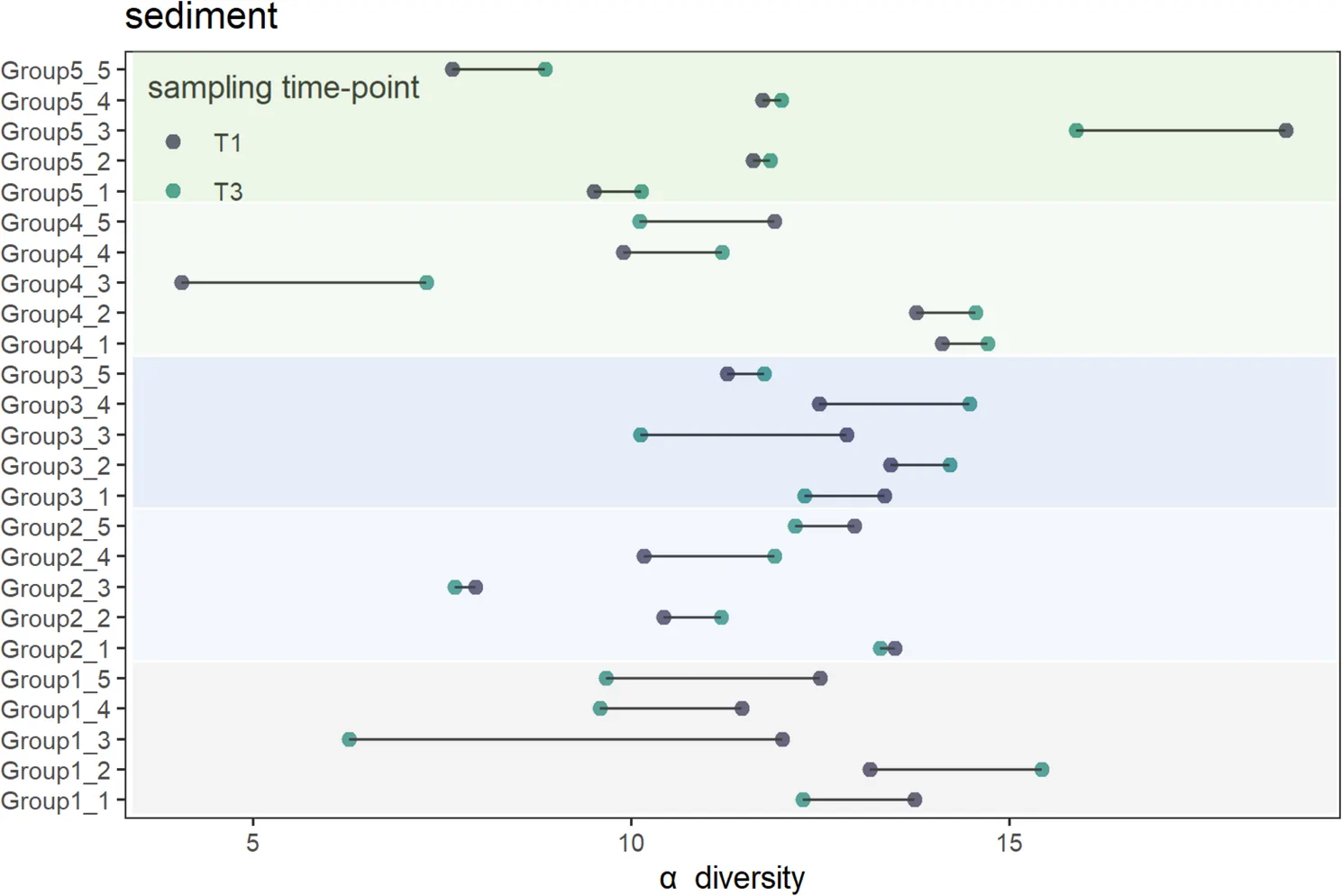
Alpha diversity (Shannon index) of sediment samples. T1 (day 5) and T3 (day 100) represent the consecutive sampling time points. Group 1 is the control group (5 ponds), Group 2 is experimental group W1 (5 ponds), Group 3 is experimental group W1-F1 (5 ponds), Group 4 is experimental group W2 (5 ponds), and Group 5 is experimental group W2-F2 (5 ponds), where F and W denote feed and water supplements, respectively

**Fig. 5:**
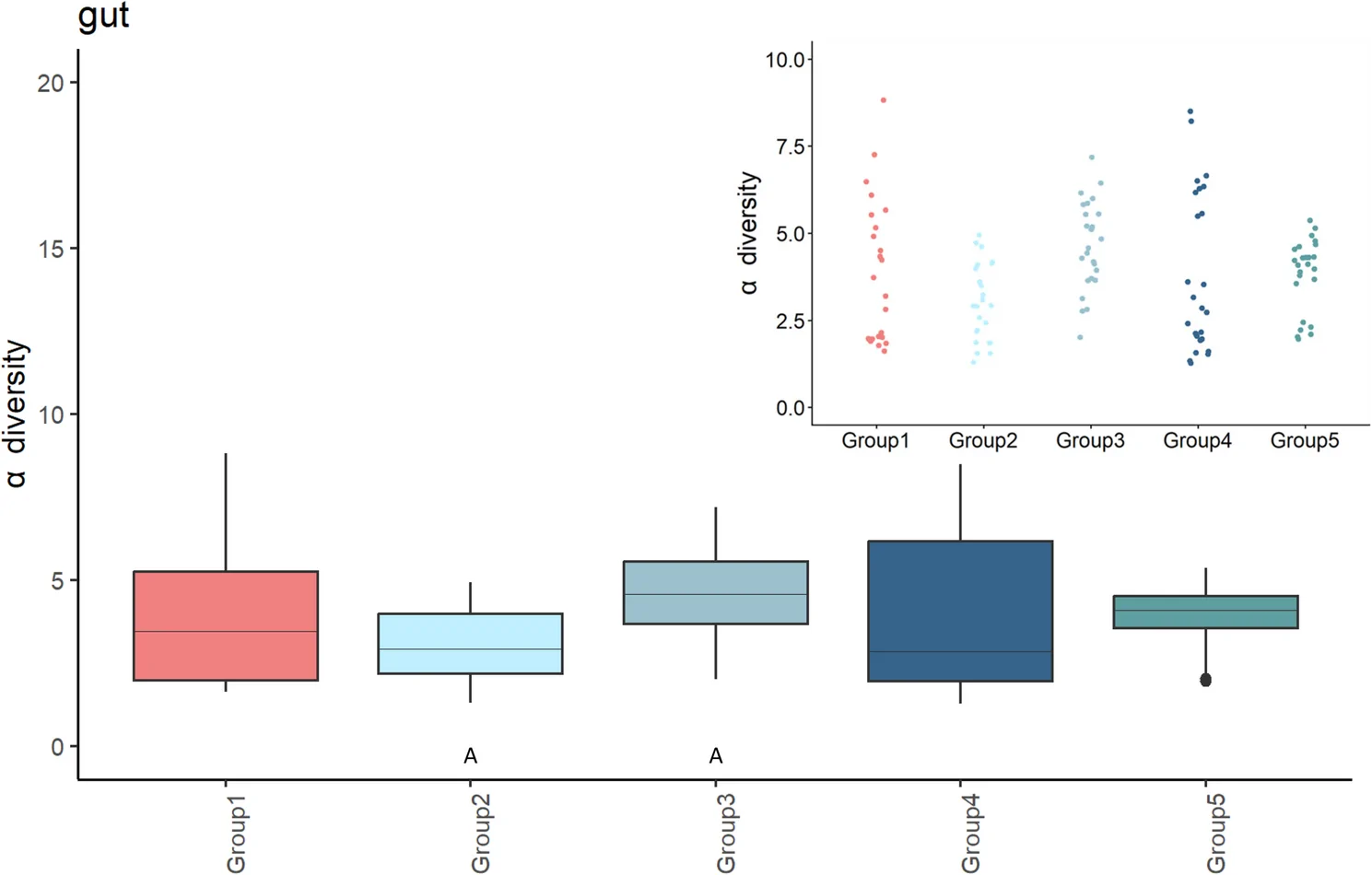
Alpha diversity (Shannon index) of gut samples from ponds and individual fish (inset). Group 1 is the control group (5 ponds), Group 2 is experimental group W1 (5 ponds), Group 3 is experimental group W1-F1 (5 ponds), Group 4 is experimental group W2 (5 ponds), and Group 5 is experimental group W2-F2 (5 ponds), where F and W denote feed and water supplements, respectively. Letters mark groups with significant (*P* < 0.05) pairwise - diversity

The median microbial diversity of the water samples (Fig. 3) at the first sampling time point (T1) was similar across all groups. However, the alpha diversity (standard deviations of 0.81 and 0.80) of the ponds supplemented with water supplemented with W1 was more uniform than that of the five control ponds (standard deviation of 2.32) and the ponds supplemented with W2 (standard deviations of 2.52 and 1.61). The dynamics of within-pond diversity exhibited a distinct pattern that differed between the control and the experimental ponds. In the control ponds, microbial diversity increased with time, whereas all the experimental points presented an initial increase in diversity from T1 to T2, followed by a decrease towards the end of the experiment (T3). Compared with the water samples, the sediment samples generally presented less pronounced changes in alpha diversity over time. Although no strong patterns emerged in the diversity attributed to the different groups, in general, the alpha diversity of the experimental groups tended to increase over time, whereas the opposite was observed for the control group (Fig. 3). When examining individual ponds serving as experimental replicates ( Fig. 4), changes in sediment diversity over time appeared more pronounced in the control ponds than in the supplemented ponds. In particular, in four out of the five control ponds, the diversity decreased within the range of 1.48 to 5.72, whereas in one pond, the opposite trend was observed. The diversity dynamics of the supplemented ponds revealed no pattern regarding an increase or decrease in diversity within each pond, with values ranging from 0.20 to 2.78 ( decreased diversity) and 0.22 to 3.24 ( increased diversity). Our analysis revealed that the diversity of the microbial communities in the fish gut (Fig. 5) was markedly lower than that in the environmental samples (water and sediment). Fish in Group 3 receiving water and feed supplementation W1-F1 exhibited slightly higher gut microbiome diversity levels (average of 4.65) than those in the other groups (3.84, 3.03, 3.82, and 3.83). However, as expressed by the within-group standard deviation of individual alpha diversities, the level of gut microbiome diversity was more similar among individuals subjected only to water supplementation in Group 2 (1.08) and water and feed supplementation with W2-F2 supplementation in Group 5 (1.04). The alpha diversity of the sequenced supplements was considerably lower than the gut and environmental diversity, with values of 0.57, 0.63, and 0.38, respectively for W1, F1, and W2-F2 (that constitute the same product supplemented in two different forms to water and feed). More specifically, W1 comprised two families with relative abundances of Lactobacillaceae (0.976) and Acetobacteraceae (0.024), F1 contained three families with relative abundances of Lactobacillaceae (0.900), Clostridiaceae (0.093), and Sporolactobacillaceae (0.007), and W2-F2 consisted of Clostridiaceae (0.909), Lactobacillaceae (0.055), and Paenibacillaceae (0.036).

### Dominant bacteria families

**Fig. 6:**
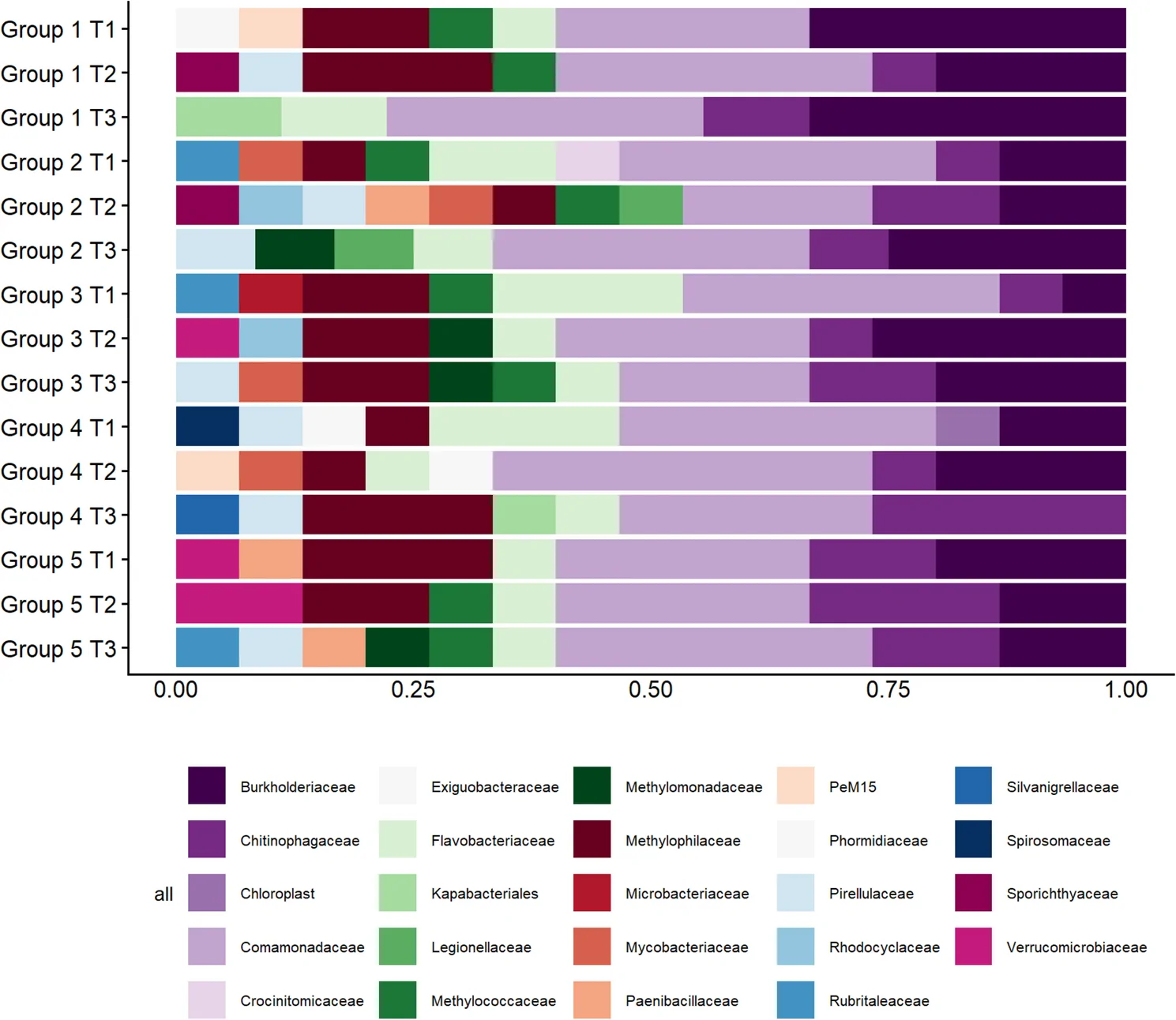
Frequencies of the three most abundant families in the water pooled over the experimental groups. T1 (day 5), T2 (day 47), and T3 (day 100) represent the consecutive sampling time points. Group 1 is the control group (5 ponds), Group 2 is experimental group W1 (5 ponds), Group 3 is experimental group W1-F1 (5 ponds), Group 4 is experimental group W2 (5 ponds), and Group 5 is experimental group W2-F2 (5 ponds), where F and W denote feed and water supplements, respectively

In water, Comamonadaceae was consistently the most abundant family under all the experimental conditions. Burkholderiaceae, Chitinophagaceae, and Methylophilaceae were also highly abundant in most ponds. Notably, the bacterial families provided in the supplements were either undetectable or of very low abundance, except for Paenibacillaceae from the W2 and F2 supplements, which was highly abundant in one pond from experimental Group 5 supplemented with W2-F2 but somewhat unexpectedly in one pond from experimental Group 3, which was not supplemented with W2 or F2 (Fig. 6).

**Fig. 7:**
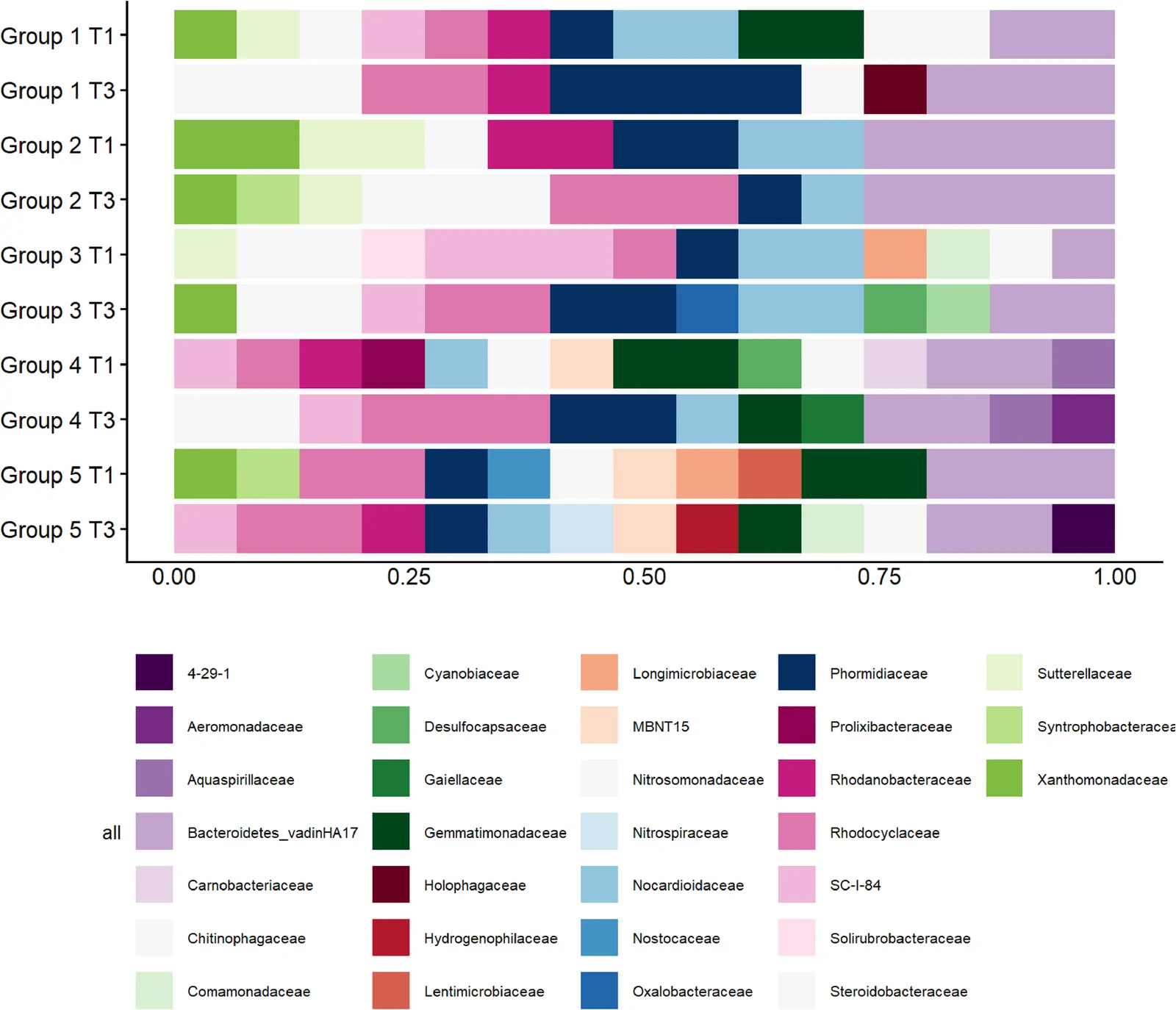
Frequencies of the three most abundant families in the sediment pooled across the experimental groups. T1 (day 5) and T3 (day 100) represent the consecutive sampling time points. Group 1 is the control group (5 ponds), Group 2 is experimental group W1 (5 ponds), Group 3 is experimental group W1-F1 (5 ponds), Group 4 is experimental group W2 (5 ponds), and Group 5 is experimental group W2-F2 (5 ponds), where F and W denote feed and water supplements, respectively

Compared to the water samples, the sediment samples presented considerably greater variation in the most abundant families across ponds. They showed a variable pattern of high abundance, where numerous families were highly, but often uniquely, represented within a single sample (Fig. 7). This may have been caused by the greater influence of spatial sampling heterogeneity within the ponds, where the water exhibited greater spatial uniformity due to increased movement within a pond. Bacteroidetes_vadinHA17 was present in most ponds, albeit not all, whereas each group (including the control) contained relatively high counts of *Bacteroidetes_vadinHA17*, *Rhodocyclaceae*, *Nocardioidaceae*, and *Phormidiaceae*. Supplementation with W2/W2-F2 resulted in more variable patterns of highly abundant bacterial families, whereas the most consistent pattern was observed in the five ponds supplemented with W1. Most of the bacterial families introduced with the supplements were not established in the sediment, with the exception of *Clostridiaceae*, which was present in almost all ponds, including the control (i.e. unsupplemented ponds), indicating that it is a naturally abundant bacterial family. The abundance of *Paenibacillaceae* supplemented with W2-F2 was detected in only one control pond and one pond supplemented with W2.

**Fig. 8:**
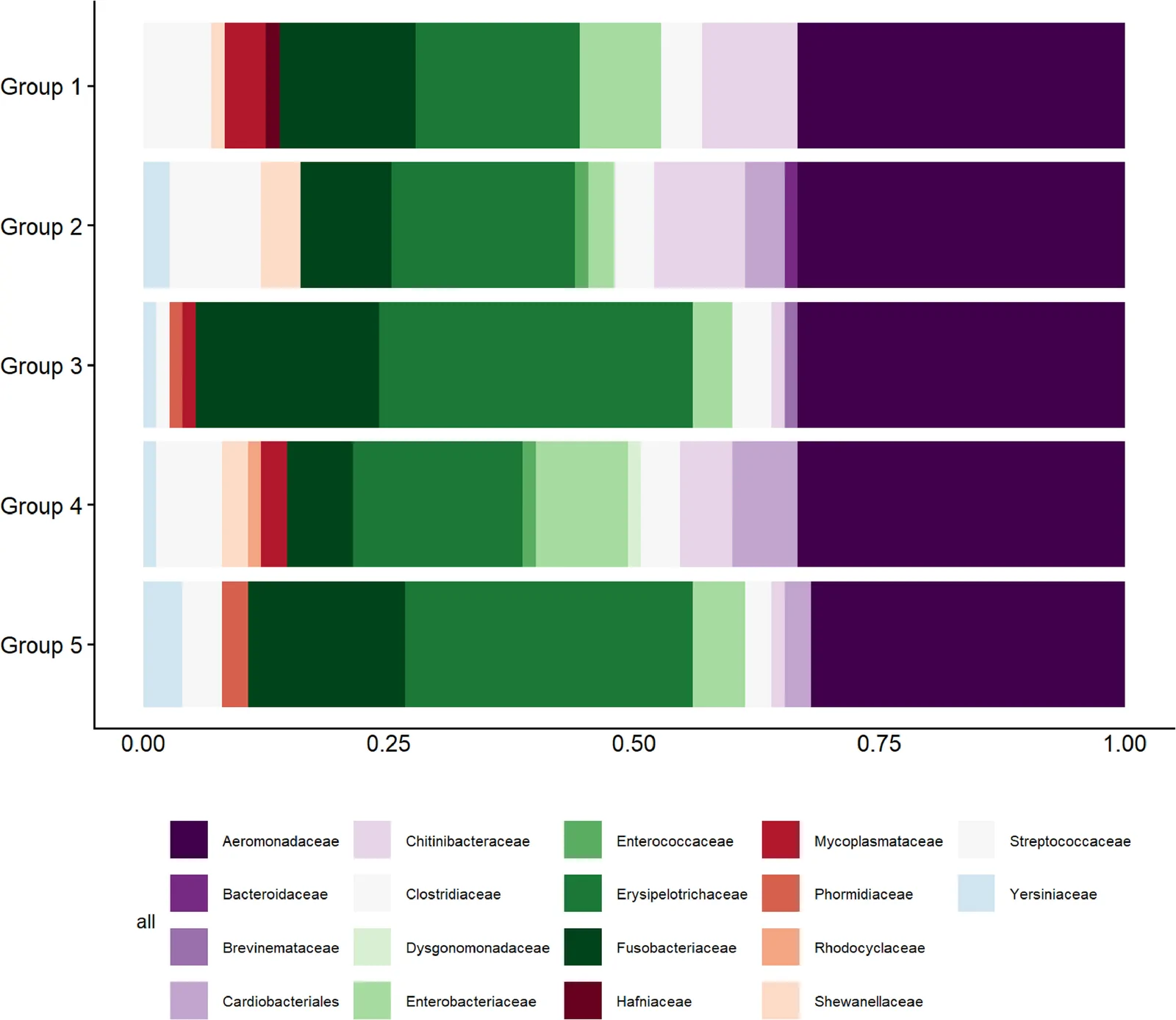
The frequencies of the three most abundant families in the fish gut pooled over the experimental groups. Group 1 is the control group (5 ponds), Group 2 is experimental group W1 (5 ponds), Group 3 is experimental group W1-F1 (5 ponds), Group 4 is experimental group W2 (5 ponds), and Group 5 is experimental group W2-F2 (5 ponds), where F and W denote feed and water supplements, respectively

In the gut, Aeromonadaceae was among the most abundant families in each fish, except for a single individual. Furthermore, Erysipelotrichaceae and Fusobacteriaceae were among the most common families, regardless of the experimental conditions (Fig. 8). Additionally, within-pond similarity among fish in terms of the most abundant families was not greater than that among fish from different ponds. With respect to the bacterial families contained in the supplements, Clostridiaceae, which were supplemented in F1 and F2, were detected in most individuals, except for nine fish, but the pattern was not related to supplementation, indicating that the family is naturally abundant in the gut, with no supplementation needed. Unexpectedly, Paenibacillaceae supplemented in F2 was present in two individuals, but none of them were supplemented with F2. The other families supplemented in the feed (Lactobacillaceae and Sporolactobacillaceae) were not present. Evidence indicates that feed supplementation has little to no effect on gut microbiome composition.

### Differences in bacterial composition

**Fig. 9:**
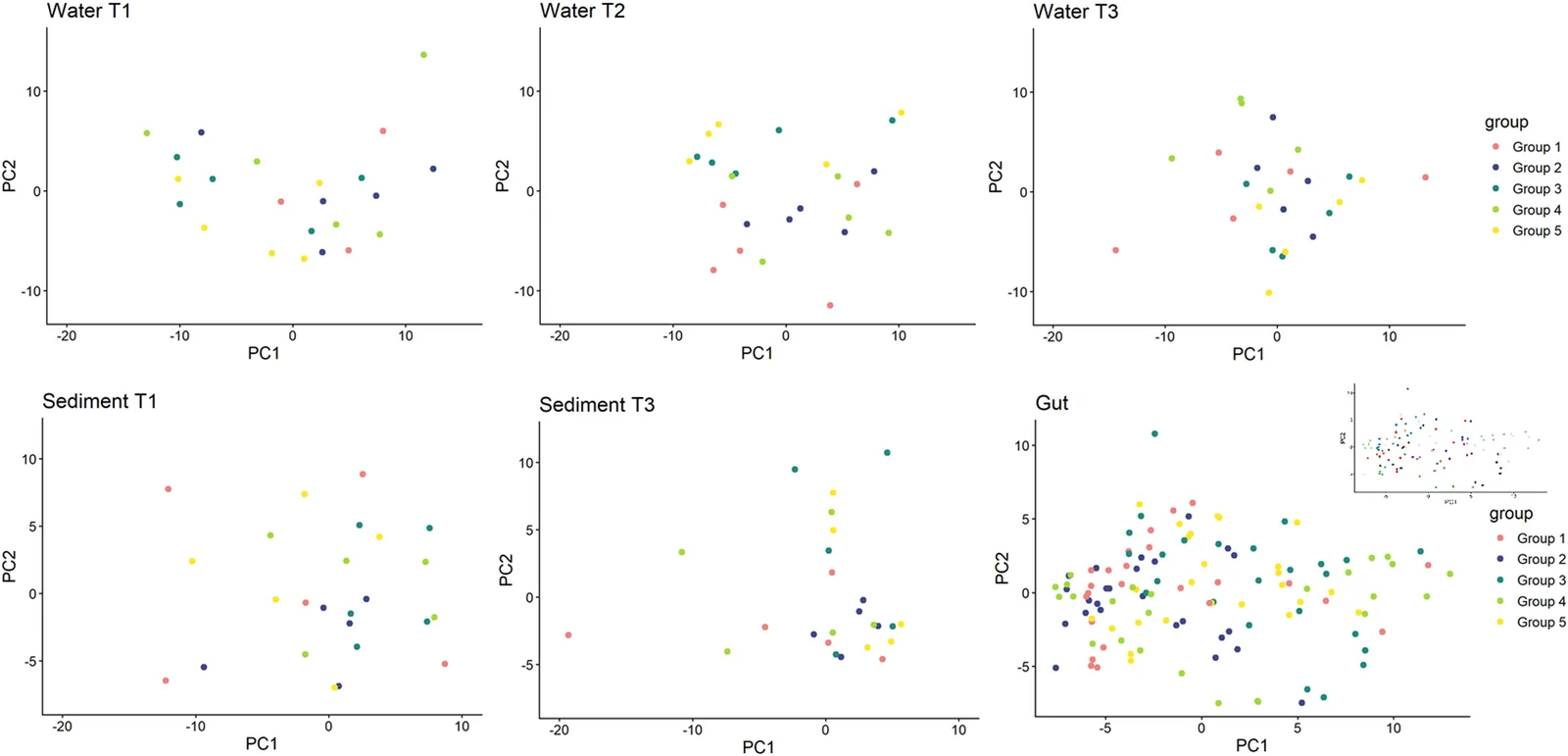
PCA representation of bacterial family abundance characteristic for each pond. T1 (day 5), T2 (day 47), and T3 (day 100) represent the consecutive sampling time points. Group 1 is the control group (5 ponds), Group 2 is experimental group W1 (5 ponds), Group 3 is experimental group W1-F1 (5 ponds), Group 4 is experimental group W2 (5 ponds), and Group 5 is experimental group W2-F2 (5 ponds), where F and W denote feed and water supplements, respectively

The next step was to consider the global pattern of all families detectable in the samples to check whether it is distinct between experimental groups. Figure 9 summarises the visual representation of the bacterial family abundances characteristic of each of the studied environments (water, sediment, and gut). Although no distinct clustering patterns related to the experimental conditions emerged, it was clear that during the experiment, both the water and sediment bacterial communities tended to converge, becoming more similar across the experimental conditions.

### Differences in the abundance of single families

As no clear pattern emerged for the overall microbial diversity, the second part of the study focused on particular families.

The analysis of the differential abundances of bacterial families in the water samples at the first sampling time point revealed no significant differences in abundance between the control and the experimental ponds, even though the T1 sample collection was one week after the first water supplementation. At the 2nd sampling time point, there were still no significantly altered abundances between the control group (Group 1) and Group 2, but in the remaining three supplemented groups, a significant increase in the abundance of Rubritaleaceae was observed. The corresponding LFCs were 6.4 (*P* = 0.003), 7.5 (*P* = 1·10^− 4^), and 7.7 (*P* = 7·10^− 5^), respectively, for Groups 3–5. Furthermore, a significant increase in Solirubrobacteraceae abundance with an LFC of 7.6 (*P* = 0.022) was detected in Group 5. However, the observed effect did not persist toward the end of the experiment, hence, at the last sampling time point (T3), which occurred 54 days after T2, the differences in the abundances of Rubritaleaceae and Solirubrobacteraceae were no longer significant. Nevertheless, the microbial community demonstrated further dynamics at the last time point, and significant decreases in the abundances of bacterial families were observed involving Carnobacteriaceae in Groups 2 and 3, with LFCs of -21.6 (*P* = 2·10^− 12^) and − 22.0 (*P* = 1·10^− 13^), respectively, Geobacteraceae (LFC=-22.7, *P* = 7·10^− 14^) in Group 2, Sphingobacteriaceae (LFC=-7.6, *P* = 0.047) in Group 3, Methylomonadaceae (LFC=-8.3, *P* = 0.007) and Moraxellaceae (LFC=-22.7, *P* = 8·10^− 14^) in Group 4, and Exiguobacteraceae (LFC=-8.8, *P* = 0.037) in Group 5. Notably, except for Methylomonadaceae, none of these families were among the most abundant, indicating that supplementation predominantly affected the less abundant families. Results of the differential abundance analysis were summarised in Supplementary table S7.

The results obtained for T3 were further refined with the metatranscriptome data, allowing for an unbiased view of both the eukaryotic and bacterial microbiomes, which were inaccessible through our 16 S rRNA gene amplicon sequencing. In total, 231,353,334 metatranscriptome reads from 533,108,406 were filtered during quality control. On average, 2,138,017 and 1,168,061 reads per pond matched the large ribosomal subunit (LSU) and small ribosomal subunit (SSU), respectively, and were taxonomically assigned. With respect to LSU-based annotations, no significant differential abundance was detected between the control and experimental ponds, presumably due to the poorer composition of the SILVA database for LSU sequences. Annotation based on SSU indicated a significant differential abundance of nonbacterial microorganisms (Supplementary table S8). Two eukaryotic genera, the monotypic fungus Linostomella sp. in Group 3 (LFC = 5.3, *P* = 2·10^− 4^) and Group 4 (LFC = 9.8, *P* = 9·10^− 4^), and the unicellular protist Dileptus sp. in Group 2 (LFC = 22.2, *P* = 1·10^− 13^), were significantly more abundant in the experimental ponds, whereas the abundance of another protist species, *Paramecium bursaria*, decreased significantly under W2 supplementation in Group 4 (LFC=-7.3, *P* = 0.045).

For the sediment samples, at the first time point, a significant increase in abundance was detected after supplementation for Group 3, with Cyanobiaceae (LFC = 8.4, *P* = 0.012), which even became one of the three most abundant families in a pond representing this experimental group. At the second time point, bacteria belonging to the Flavobacteriaceae family (LFC=-7.1, *P* = 0.039) significantly decreased in abundance in the experimental ponds in Group 3, whereas the abundances of Streptococcaceae (LFC = 22.9, *P* = 1·10^− 16^) and Aquaspirillaceae (LFC = 25.0, *P* = 6·10^− 23^) significantly increased in Group 4. The latter was one of the most abundant families in the two ponds in this group.

In the fish gut, supplementation led to an increase in only a few bacterial families compared to that in the control ponds. Supplementation with W1-F1 (Group 3) resulted in a significant increase in the abundance of Weeksellaceae (LFC = 6.5, *P* = 2·10^− 7^), Geobacteraceae (LFC = 6.8, *P* = 0.001), and Methylomonadaceae (LFC = 8.9, *P* = 4·10^− 14^). Water supplementation with W2 in Group 4 led to a significant increase in the abundance of Erysipelotrichaceae (LFC = 2.0, *P* = 0.008), which was among the most abundant families in the gut microbiomes of many fish in all the experimental ponds. Furthermore, in Group 4, we observed increased abundances of the Yersiniaceae (LFC = 4.1, *P* = 0.013), Enterobacteriaceae (LFC = 6.5, *P* = 1·10^− 3^), and Cardiobacteriales (LFC = 2.8, *P* = 0.010) bacterial families, which are common in the gut microbiomes of some fish from experimental ponds. Supplementation with W2-F2 in Group 5 affected the abundance of these four families. A significant increase was detected for: Erysipelotrichaceae (LFC = 2.4, *P* = 2·10^− 5^) which is a commonly abundant family in fish from all ponds, another commonly abundant family, Cardiobacteriales (LFC = 2.4, *P* = 0.023), and Methylomonadaceae (LFC = 7.2, *P* = 9·10^− 7^), which was not among the most highly abundant. Conversely, the abundance of Streptococcaceae, which was highly abundant in all ponds, significantly decreased (LFC=-3.6, *P* = 0.032) after W2-F2 supplementation in Group 5.

Notably, Group 2 supplemented with W1 showed no significant alterations in bacterial family abundance in either the sediment or gut environment.

Within each pond, regardless of the supplementation (W1 or W2), water and sediment abundance of the families that were reported by the supplements’ manufacturers (Lactobacillaceae, Bifidobacteriaceae, Carnobacteriacea, Streptococcacea, and Rhodobacteraceae) was not significantly altered over the course of the experiment (Supplementary tables S17-19). Even in control, unsupplemented ponds, as demonstrated by the linear regression coefficients calculated within each supplementation unit not significantly different from zero. The only significant change was decrease of Rhodobacteraceae abundance in sediment in the control ponds that were not supplemented. Moreover, Nitrobacteraceae claimed to be contained in F1 were not detected neither in supplement, nor in unsupplemented ponds.

### Water quality measurements

During the experiment, all monitored water quality parameters remained within the acceptable range for the common carp and although the chemical compounds varied within ponds over time, there weren’t any significant differences between the experimental groups within sampling times. The only exception was a significantly higher PO_4_ concentrations in Group 2 (*P* = 0.0259) and Group 5 (*P* = 0.0142) as compared to Group 1, during the last sampling. This suggests that the supplementation had a relatively subtle impact on the pond chemistry.

### Fish survival rates and body measurements

**Fig. 10:**
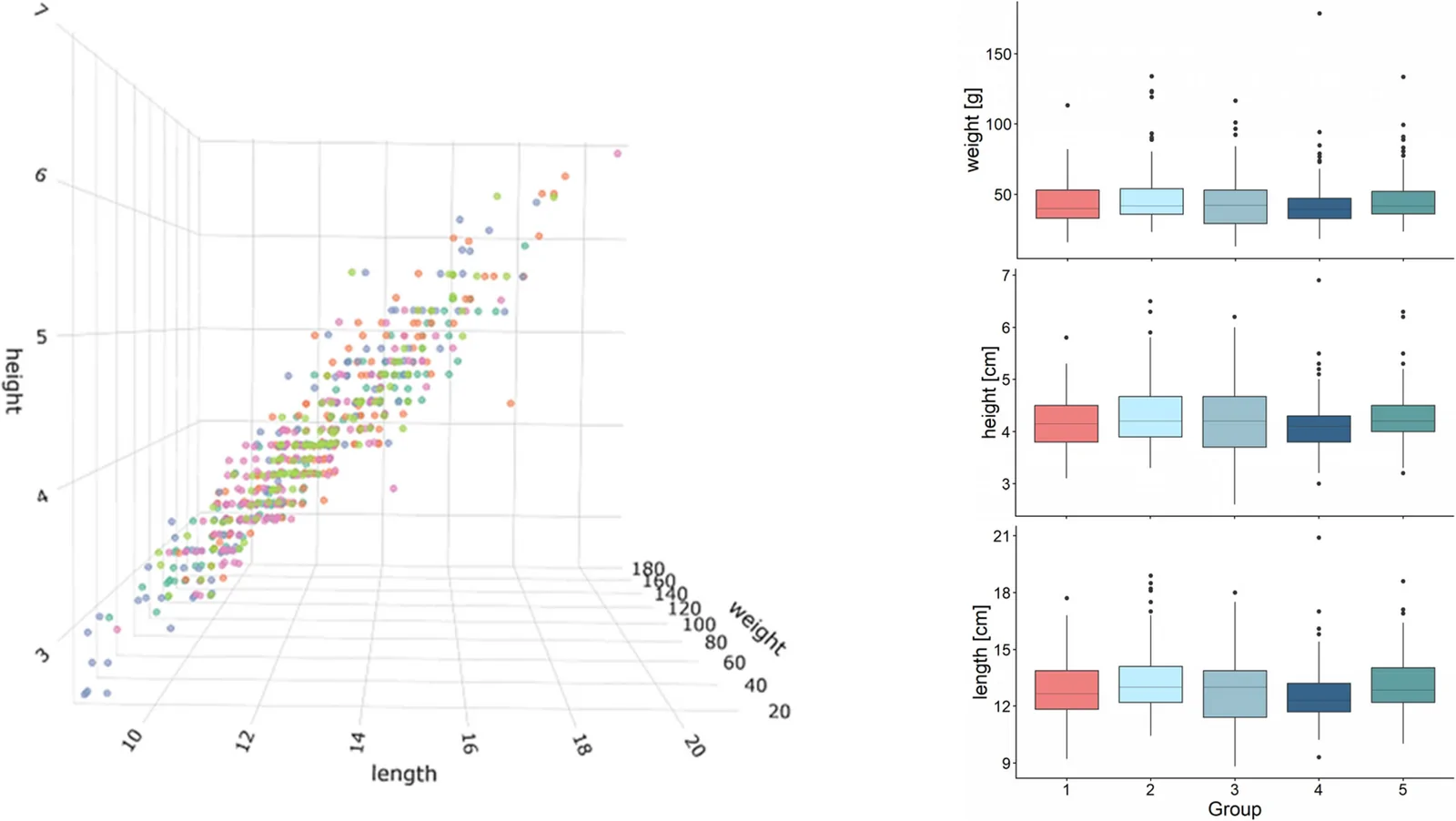
Fish body measurements. Group 1 is the control group (5 ponds), Group 2 is experimental group W1 (5 ponds), Group 3 is experimental group W1-F1 (5 ponds), Group 4 is experimental group W2 (5 ponds), and Group 5 is experimental group W2-F2 (5 ponds), where F and W denote feed and water supplements, respectively

All pairwise differences between experimental groups in the estimated fish survival rates (Supplementary table S20) were significant. The highest survival rate of 62%±0.11 was observed for Group 5 and the lowest (54%±0.15) for Group 2, while the control group survival rate of 58%±0.04 was intermediate. For each of the three body measurements depicted in Fig. 10, the differences between the experimental groups were significant, varying between *P* = 0.0105 for weight and *P* = 7·10^− 5^. For height. Specifically, fish in Group 2 were significantly larger than those in the control group (on average: 4.39 g heavier, 0.42 cm longer, and 0.20 cm higher), but also larger than those in experimental Groups 3 and 4. However, the within-group variability of fish measurements between ponds was much more significant than the variability between groups.

The common feature of the penalised linear regression models was their poor fit, expressed by R^2^ values of 0.22 for weight, 0.10 for height, and 0.19 for length. The highest R^2^ (0.22) and the largest number of selected bacterial families (23) were identified for the weight. The highest positive effect of 8.3 g weight associated with an increase in one abundance unit was estimated for the order Flavobacteriales without a particular family assigned, and the highest negative effect of a 5.9 g decrease in weight was associated with an increased abundance of Silvanigrellaceae. In terms of length, 13 bacterial families were selected, with the highest positive effect of 0.6 cm associated with the increased abundance of Nocardiaceae and the highest negative effect of 0.9 cm, with increased abundance of Silvanigrellaceae, the same family that also negatively influenced weight. The abundance of Nocardiaceae was also highly positively associated with weight. For height, only six families were selected, albeit with overall low effects, ranging from 0.02 cm (Sporomusaceae) to -0.05 cm (Phormidiaceae).

## Discussion

Real-life conditions provide more accurate insights into microbial products perform in actual aquaculture settings. Our study characterised three different environments, i.e., water, sediment, and fish gut, to evaluate the efficacy of effective microorganism supplementation in modifying the microbial composition and improving the growth of common carp in earthen ponds. We also noted discrepancies between the expected and actual composition of the supplements and did not observe a clear influence of supplementation on the abundance of the intended bacterial families.

It’s essential to note that the commercial products investigated in our study involved supplementation with Effective Microorganisms, which fundamentally differs from probiotic supplements. Unlike probiotics, EM products contain a mixture of microorganisms that are not necessarily alive and their microbial composition is not strictly defined. In light of this, the observed discrepancies between the manufacturer’s claims and the actual microbial composition of the supplements are less surprising, but provide implication on the impact of EM based supplementation. Most changes over time were observed for water, in contrast to the control ponds, where natural diversity increased during the experiment. In all supplemented groups, the diversity dynamics of the water microbiome were the same after the initial increase (which may have been due to supplementation), and there was a decrease in diversity towards the end of the experiment. This suggests that although the introduced bacterial families may not have directly established themselves, they indirectly influenced the ecosystem by altering the abundance of other families, which, however, had only a temporary effect. Additionally [34], reported an increase in water alpha diversity in untreated and control ponds and changes in alpha diversity in supplemented ponds, albeit following a different pattern than that observed in the present study. As demonstrated by PCA, not only were no distinct bacterial communities established by supplementation but also for water and sediment, during the course of the experiment, the microbiomes tended to converge, that is, they were more similar across the experimental conditions and the control. Additionally, the abundance of families that were most highly represented in each environment was not markedly influenced by supplementation.

Although it could have been expected that water and sediment are ecologically quite similar environments, the former is much more unified that leads to more consistency between samples, while sediment is more constrained to sampling sites – therefore no dominant families can be identified in sediment. We observed that gut microbiome is quite resilient to changes due to feed supplementation. The presence of Clostridiaceae in most individuals regardless of supplementation suggests it is a natural inhabitant of the gut, and the lack of establishment of other supplemented families supports the idea that feed supplementation has no significant impact on gut microbiome community structure.

Still, we observed changes in abundance of specific bacterial families. Species from the Rubritaleaceae family, whose abundance increased in experimental Groups 3–5, show antioxidative activities [35] and are involved in polysaccharide degradation [36]. The increased abundance of Solirubrobacteraceae was somewhat unexpected since the family is typical of soil environments and may represent a sampling or sequencing artefact. The abundance of Carnobacteriaceae in water significantly decreased at the end of the experiment in Groups 2 and 3 (both supplemented with F1), which represents the group of lactic acid bacteria that, in aquaculture, are predominantly found in the distal intestine of fish and exhibit a positive effect on digestion [37]. However, species representing this family have also been found in lakes. This family has also been investigated in the context of food degradation, preservation, and oil degradation in wastewater processing [38]. In addition, the abundance of Geobacteraceae was significantly altered by the supplementation. In water, it decreased at the end of the experiment in Group 2, whereas in the gut, its abundance was higher after supplementation with F1. Species from this family predominantly reside in sediment, where they induce the release of soluble Fe(II) from insoluble Fe(III) into water, which may further stimulate the growth of other microorganisms [39]. Sphingobacteriaceae, which were less abundant in the water of experimental Group 3, originally represented the soil-inhabiting family but was also reported in aquaculture as containing species that metabolise cellulose, chitin, collagen, and nitrogen compounds in water [40]. Flavobacteriaceae, which significantly decreased in abundance in sediment after W1-F1 supplementation in Group 3, are common in planktonic freshwater communities [41] and are considered potentially pathogenic. Members of this family often metabolise large molecules, such as polysaccharides and proteins [42]. Weeksellaceae, whose abundance was significantly increased in Group 3, represents an unwanted pathogenic family that promotes inflammation and disrupts the formation of intestinal villi in fish [43]. Erysipelotrichaceae, which is considered an unwanted, potentially pathogenic family, was not only among the most abundant in the fish gut, but also its gut abundance was significantly increased after F2 supplementation. The same was true for the significant increase in the pathogenic Yersiniaceae. This observation contradicts the recent results obtained for shrimp *Penaeus vannamei* [44] and largemouth bass [45]. In contrast, Group 4 supplemented with W2-F2 presented a significant increase in the beneficial family Enterobacteriaceae, which is considered a probiotic supplement (see, for example [13], , and a significantly decreased abundance of the Streptococcaceae family, which contains fish pathogenic species such as *Lactococcus piscium* [38].

Even though no significant changes in water or sediment microbial communities occurred due to supplementation investigated in our study, the weight, length, and height of the fish in Group 2 (water supplemented with W1) had was significantly higher as compared to the control group, whereas those in the other experimental groups did not significantly differ from the control. Interestingly, measurements for Group 5 were not significantly different from those in Group 2 which correlates with the significantly increased PO_4_ concentrations in water observed in both groups at the last sampling. This suggests that the microbial compositions specific to W1 and W2-F2 supplementations altered biochemical processes in water, leading to increased phosphate concentrations, thereby creating a phosphate-rich environment that may promote fish growth. However recent literature did not observe an effect of phosphate pond fertilisation on the growth performance of common carp [46]. Moreover, the literature shows mixed results regarding the effects of effective microorganisms compounds on the growth performance of carp. In a 31-day feeding experiment [47], did not observe a significant effect of feed supplementation with commercial supplements on carp growth performance, while a positive effect of commercial microbial products on growth was reported by [48]. In contrast, positive effects of targeted supplementation with specific genera have been reported. Supplementation with Lactococcus has been shown to improve the growth performance of common carp [11, 49]. A positive effect of Lactobacillus supplementation was reported by [50, 51, 52]. Conversely, in a field experiment reported by [17], no significantly better carp growth performance was observed with an experimental diet supplemented with Lactobacillus species. Additionally, experimental diets with selected Bacillus species had a positive effect on carp growth [53, 54]. These authors suggested that the effect was due to the positive influence of supplementation on glucose absorption and overall metabolism of the carp, as well as better health status due to improved immune resistance of the fish.

This discrepancy in the literature can be attributed to two main reasons. First, applying experimentally prepared diets containing a specific composition of species at a predefined concentration may substantially differ from supplementation with commercially available products, for which the microbial composition is often not well defined and not specifically controlled in each particular sample. This problem was demonstrated in our study, where sequencing results of commercial supplements did not detect all the microorganisms that were listed as the designated components, indicating that some of the genera might be present in very low abundance under the technical detection threshold. The second limitation is that most of the abovementioned studies (with the exception of [17]) were conducted under experimental conditions in tanks. In particular, in the context of fish growth, it is expected that such environmentally unified conditions will strongly deviate from the situation in the field. Although we are not aware of systematic comparisons of the tank and experimental growth performance of fish, owing to environment‒microbiome‒phenotype interactions that are not fully modelled in tank-based experiments, the experimental results may not be directly transferable to the field. Metatranscriptomic analysis revealed a significant increase in the abundance of protozoan species ( especially, *Linostomella* sp. and *Paramecium bursaria*, which serve as water quality indicators. This suggests that despite the absence of clear patterns in bacterial composition and single-family abundance, the overall water quality improved by the end of the experiment.

Given our observation that supplementation with microbial products had no effect on the bacterial community composition, we also estimated the effects of particular families on fish growth performance. The highest significant positive effect on weight with increasing abundance of Flavobacteriales may be related to the fact that members of this typically highly abundant family can “assist” in probiotic supplementation by degrading polysaccharides [55]. Conversely, the highest negative effect on fish weight and length with increasing abundance was estimated for Silvanigrellaceae, which has not been characterised in the context of aquaculture. Surprisingly, a positive effect on weight and length was estimated for the increased abundance of the Nocardiaceae family. Surprisingly, some Nocardia species cause nocardiosis, a serious disease in aquaculture. However, this family also contains species that play a role in organic matter turnover and degradation of xenobiotics, which may have beneficial effects on fish (summarised in [56]).

One limitation of our study was the fact that the stability of the microbial composition of the supplements was not monitored during the course of the experiment. Moreover, our experimental design lacked groups with feed supplementation only (say, F1 and F2), which exceeded the capacities of our experimental facility, as we prioritised the importance of having replications. For future research, follow up experiments that target metabolites and immune resistance would form a logical extension.

## Conclusion

Although effective microorganism supplementation with a commercial product did not significantly modify the water, sediment, or gut microbiome of the fish, an increase in the final weight was still observed in fish reared in ponds supplemented with W1. This experimental group (Group 2) was distinct from the other experimental conditions because it presented the lowest and most consistent alpha diversity of water and the gut across ponds and appeared to be the most stable in terms of diversity across the course of the experiment. Based on the results obtained for this group, we hypothesize that water supplementation promotes a stable microbial environment, which in turn positively impacts fish health.

## Supplementary Information

Supplementary Material 1.

## Data Availability

The 16 S rRNA Gene Sequencing Data is available in the NCBI BioProject repository. It can be accessed using the following DOI or accession ID: https://identifiers.org/ncbi/bioproject: PRJNA1315210.The Metatranscriptome Sequencing Data is also available in the NCBI BioProject repository. This dataset can be accessed using the same DOI or accession ID: https://identifiers.org/ncbi/bioproject: PRJNA1315210.The Microbial Community Composition, Differential Abundance, and Fish Phenotypes are available as Supplementary Material.

## Abbreviations

ANOVA: Analysis of variance
ASV: Amplicon Sequence Variants
CLR: Centered Log-Ratio
LASSO: Least Absolute Shrinkage and Selection Operator
LFC: Log_2_FoldChange
LSU: Large ribosomal subunit
PCA: Principal Component Analysis
MCE: Mixed cellulose ester
SSU: Small ribosomal subunit
